# Characterization of Pipefish Immune Cell Populations Through Single-Cell Transcriptomics

**DOI:** 10.3389/fimmu.2022.820152

**Published:** 2022-01-27

**Authors:** Jamie Parker, Naomi Croft Guslund, Sissel Jentoft, Olivia Roth

**Affiliations:** ^1^ Marine Evolutionary Ecology, GEOMAR Helmholtz Centre for Ocean Research Kiel, Kiel, Germany; ^2^ Marine Evolutionary Biology, Christian-Albrechts-University, Kiel, Germany; ^3^ Centre for Ecological and Evolutionary Synthesis, Department of Biosciences, University of Oslo, Oslo, Norway; ^4^ Department of Immunology, Institute of Clinical Medicine, University of Oslo, Oslo, Norway

**Keywords:** single-cell transcriptomics, pipefish, immunity, cell profiling, immune cell

## Abstract

Teleost adaptive immune systems have evolved with more flexibility than previously assumed. A particularly enigmatic system to address immune system modifications in the evolutionary past is represented by the Syngnathids, the family of pipefishes, seahorses and seadragons. These small fishes with their unique male pregnancy have lost the spleen as an important immune organ as well as a functional major histocompatibility class II (MHC II) pathway. How these evolutionary changes have impacted immune cell population dynamics have up to this point remained unexplored. Here, we present the first immune cell repertoire characterization of a syngnathid fish (*Syngnathus typhle*) using single-cell transcriptomics. Gene expression profiles of individual cells extracted from blood and head-kidney clustered in twelve putative cell populations with eight belonging to those with immune function. Upregulated cell marker genes identified in humans and teleosts were used to define cell clusters. While the suggested loss of CD4+ T-cells accompanied the loss of the MHC II pathway was supported, the upregulation of specific subtype markers within the T-cell cluster indicates subpopulations of regulatory T-cells (*il2rb*) and cytotoxic T-cells (*gzma*). Utilizing single-cell RNA sequencing this report is the first to characterize immune cell populations in syngnathids and provides a valuable foundation for future cellular classification and experimental work within the lineage.

## Introduction

The vertebrate immune system has evolved into an extremely diverse, layered network of defense mechanisms capable of combatting a wide variety of agents on a specific (adaptive) and generic (innate) level (1). The first adaptive immune system evolved in the descendants of the ray-finned fishes (Teleostei) ~450 million years ago (2). Teleosts comprise a diverse group of fishes made up of over 30,000 species, ranging in morphology, physiology and immune system constituents (3–7).

Among them, representatives of the syngnathids (seahorses, pipefishes and seadragons) are conceivably the most morphologically bizarre fishes, boasting the unique evolution of male pregnancy (8–10). The peculiarities extend to the syngnathid immune system, with *Syngnathus* and *Hippocampus* missing a functional Major Histocompatibility Complex II (MHC II) pathway (4, 11). Until recently this pathway was thought to be synonymous across vertebrates, it has been postulated that the evolution of full pregnancy with placenta-like structures in syngnathids may have been facilitated by this adaptive immune rearrangement (4). Yet, the presence of a fully functional MHC II repertoire in other pipefish species (e.g., *Nerophinae*), with basal male pregnancy without placenta-like structures, presents the lineage as an intriguing subject for immune and evolutionary studies. These immunological adaptations within the group were suggested to have coevolved with male pregnancy permitting immunological tolerance towards the semi-allogenic embryo (4, 12). Whilst recent studies have helped elucidate the syngnathid immune networks and processes, the evolutionary repercussions of such immune deficiencies in the immune cell populations remain unknown, with no established immune cell characterization of any syngnathid species.

Assessing immune cell populations historically relied on established cell surface markers receptive to specially designed antibodies, a practice almost exclusively reserved for model organisms such as humans and mice (13, 14). The advent of single-cell mRNA sequencing (scRNA-seq), the practice which facilitates the expression assessment of individual cells (15), is less restricting than using pre-designed antibodies. It can thus be applied in non-model species. Combined with advanced cell separation techniques such as Drop-seq microfluidics (16), scRNA-seq can characterize thousands of cellular gene expression profiles, from which cell type and functional groups can be deduced.

A number of teleost studies have utilized cell isolation and single cell transcriptomics to assist with the identification of brain (17, 18), kidney (19), intestine (20) and embryonic (21, 22) derived cell populations. Moreover, further work identifying teleost immune cell repertoires in a number of model fish species (23–28), have established a catalogue of putative immune cell markers that extend beyond those used in mammalian cell identification.

The immune related genomic rearrangements that have arisen within the syngnathid fish group provide an exciting opportunity to explore the immune cell evolutionary repercussions which may have occurred due to immune pathway loss. Moreover, the discovery of immune cell type identifiers could help lay the foundation for future immunological experiments and characterizing studies in the future. By employing Drop-seq micro-fluidics and scRNA-seq this study’s aim was to carry out the first characterization of the broadnosed pipefish (*Syngnathus typhle*) immune cell repertoire. Genomic alterations have resulted in the loss (*aicda*, *ciita*) or functional redundancy (*cd74*) of several MHC II pathway related genes (4). Hence, observed immune cell population distinctions were be expected when comparing pipefish with other teleosts, such as the absence of the MHC II related CD4^+^ T-cell subset. Furthermore, this investigation wanted to determine whether this adaptive immune system absence could have given rise to alternative or compensatory innate immune cell types that adopted MHC II’s functional role.

## Materials and Methods

### Ethics Statement

All aquaria set-ups and dissection methods meet the guidelines issued by the Ministerium für Energiewende, Landwirtschaft, Umwelt, Natur und Ditgitalisierung (MELUND) (Permit number V 242 – 57983/2018) and are in accordance with German animal welfare law.

### Animals

Aquaria-bred *Syngnathus typhle* reared in the GEOMAR aquaria facilities were transported to the Centre of Ecological and Evolutionary Synthesis in Oslo, Norway. Fish were kept under the standard conditions used by Beemelmanns and Roth (29). All fish were fed live and frozen mysids twice a day and fish were starved for 24 hours prior to dissection.

### Tissue Dissection and Dissociation

Three fish were used for this investigation with each of their length and weight recorded prior to dissection (length: 20.42, 25.59 and 23.00 cm | weight: 2.2, 2.35 and 2.12g). All three fish were euthanized with an MS-222 overdose (500 mg/l, Sigma-Aldrich) prior to blood extraction. Blood was suspended in 1x PBS/0.01% BSA after massaging sufficient blood from the severed tail vein. Head kidney was removed and placed in a petri dish containing 1x PBS/0.01% BSA medium and a 35-40µm cell strainer, before carefully massaging cells through the filter using a syringe plunger. Both blood and head kidney cells were centrifuged at 300 x g for 5 min at 4°C and re-suspended in 1ml 1x PBS/BSA (0.01%). All cells were kept on ice for the duration of the protocol. Cell sample concentration and quality were assessed under the stereomicroscope and diluted until cell concentrations met the required 200 cells/µl needed for microfluidic processing.

### Microfluidic Cell Capture (Drop-Seq)

This investigation adopted the original Drop-Seq methodology set out by Macosko, Basu (16) and its subsequent amended protocol (v3.1) with useful advice from Evan Macosko, Melissa Goldman and Steve McCarroll (URL:dx.doi.org/10.17504/protocols.io.mkbc4sn). Individual suspended cells and micro-particle beads were coalesced within a singular nanoliter oil droplet (80 µm) using the microfluidic droplet generator (Dolomite, UK). Beads are equipped with oligonucleotides consisting of four sections: (1) priming site, (2) “cell barcode”, (3) Unique Molecular Identifier (UMI) and (4) reverse transcription primer (30-bp oligo dT sequence). Each bead possesses multiple primers (10^8^) with identical barcodes but unique UMIs for downstream identification.

Each cell is lysed following successful droplet formation stimulating mRNA hybridization with the bead primers and the formation of single-cell transcriptomes attached to microparticles (STAMPs). STAMP reverse transcription is then carried out prior to cDNA amplification and Tn5-mediated tagmentation. To facilitate the multiplexing of multiple cell populations within the same sequencing library, unique sample barcodes were incorporated within the adapter primers during the post-tagmentation PCR procedure.

### scRNA-Seq and Gene Quantification

PCR amplification success was assessed on the Agilent BioAnalyzer High Sensitivity Chip (Agilent Technologies, Oslo, Norway) prior to library preparation. Library preparation and high output paired-end sequencing were carried out at the Norwegian Sequencing Centre (Illumina, NextSeq500, 75bp) (NSC; www.sequencing.uio.no), University of Oslo, Norway. A custom adapter sequence (Read 1, 20bp, GCCTGTCCGCGGAAGCAGTGGTATCAACGCAGAGTAC) and standard Illumina adapter sequence (Read 2, 60bp) were used. Using the Drop-Seq Core Computational Protocol (v2.0.0) (30) resultant reads were mapped to the *S. typhle* genome using STAR (31) following quality checks (FastQC v0.11.9 and MultiQC v1.9) (32, 33) and adapter removal. A digital expression matrix was constructed exhibiting transcript number (per gene, per cell) by grouping all reads by the cell barcode and UMI for each gene. The first 600-5,000 STAMPS, organized into decreasing read number order, from each sample were used for downstream analysis. STAMP number was dependent on the sample size.

### Cell and Gene Filtering

Data from all blood and head kidney samples was merged in preparation for gene expression analysis, utilizing the R package Suerat (v4.0.3) to create a singular Seurat object. To remove partially sampled/dying cells and potential doublets/multiplets, detection parameters were set only to include cells with > 150 genes and < 1300 genes and 3000 molecules (**Supplementary Figure 1**). These guidelines are supported by the clustering analysis tutorial (34) and previous fish species utilizing the same methodology (23). Sequencing information and cell numbers per sample can be found in the **Supplementary Material** (**Table S1**).

### Cell Clustering

Expression count data was scaled and normalized across all cells by library size and then log2 transformed (Seurat; “LogNormalize” method), prior to principal component analysis (PCA) of the 2,000 most variably expressed genes. Heatmaps and elbow plots were used to assess heterogeneity amongst PCs and to determine the optimal number of PCs to carry forward for further analysis (**Supplementary Figures 2, 3**). Shared nearest neighbor (SNN) modularity clustering was implemented using the Seurat “FindCluster” extension (PCs 1:23, resolution 0.35), followed by the use of Uniform Manifold approximation and projection (UMAP) for cluster visualization (35).

### Differential Gene Expression Analysis and Functional Identification

Differential gene expression analysis was carried out on genes that were expressed in ≥ 25 cells within a cluster, using the “Findmarkers” Seurat extension, which is based on the Wilcoxon rank sum test. *Post-hoc* statistical testing was carried out using the Bonferroni multiple comparison correction. The most influential differentially expressed genes for each cluster were extracted and genes with an adjusted *p* > 10^-50^ were considered for cell marker assignment and cell type classification. Annotated gene functions were deduced through independent literature searches, utilizing the Universal Protein Knowledgebase (UniProt) (36) and The Human Protein Atlas v20.1 (37).

## Results

**Figure 1:**
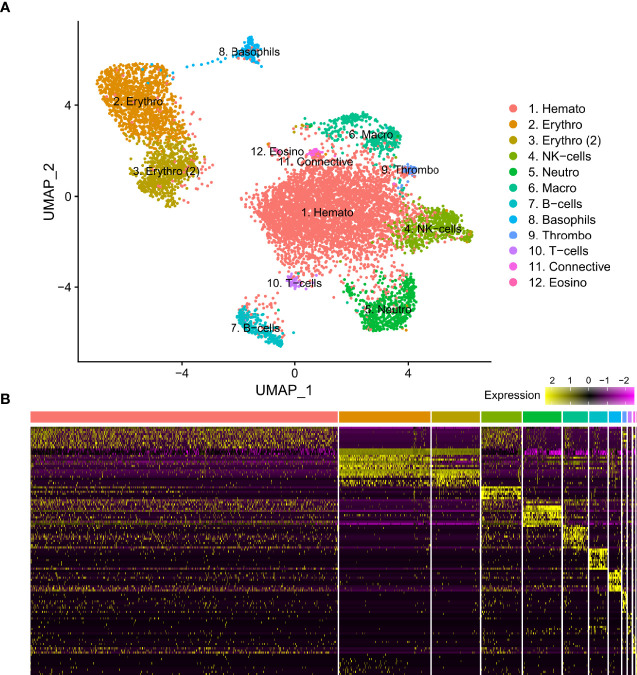
**(A)** Visual representation of isolated blood and head kidney extracted cell types of *Syngnathus typhle* using Uniform manifold approximation projection (UMAP). Cell clusters characterized by differentially expressed gene markers associated with specific cell sub-types. All markers associated with immune function previously reported in humans and/or fish species. Two erythrocyte clusters are represented with the second highlighted as such (2). Full names for all clusters are as follows: 1. Hematopoietic cells, 2 and 3. Erythrocytes, 4. Natural killer cells, 5. Neutrophils, 6. Macrophages, 7. B-cells, 8. Basophils, 9. Thrombocytes, 10. T-cells, 11. Connective tissue cells, 12. Eosinophils. **(B)** Differential gene expression heatmap highlighting top 10 marker genes, with genes representing rows and cells representing columns.

From three *S. typhle* pipefish individuals a total of 12129 genes across 8658 cells were accrued following filtering and quality control measures. 2196 cells were derived from blood and 6462 were derived from the head kidney. UMAP projection helped distinguish 12 distinct cell type clusters characterized using shared gene expression profiles (**Figure 1**). Clusters ranged in size from 12 to 4508 cells.

### Hematopoietic Cells, Erythrocytes and Connective Tissue Cells

**Figure 2:**
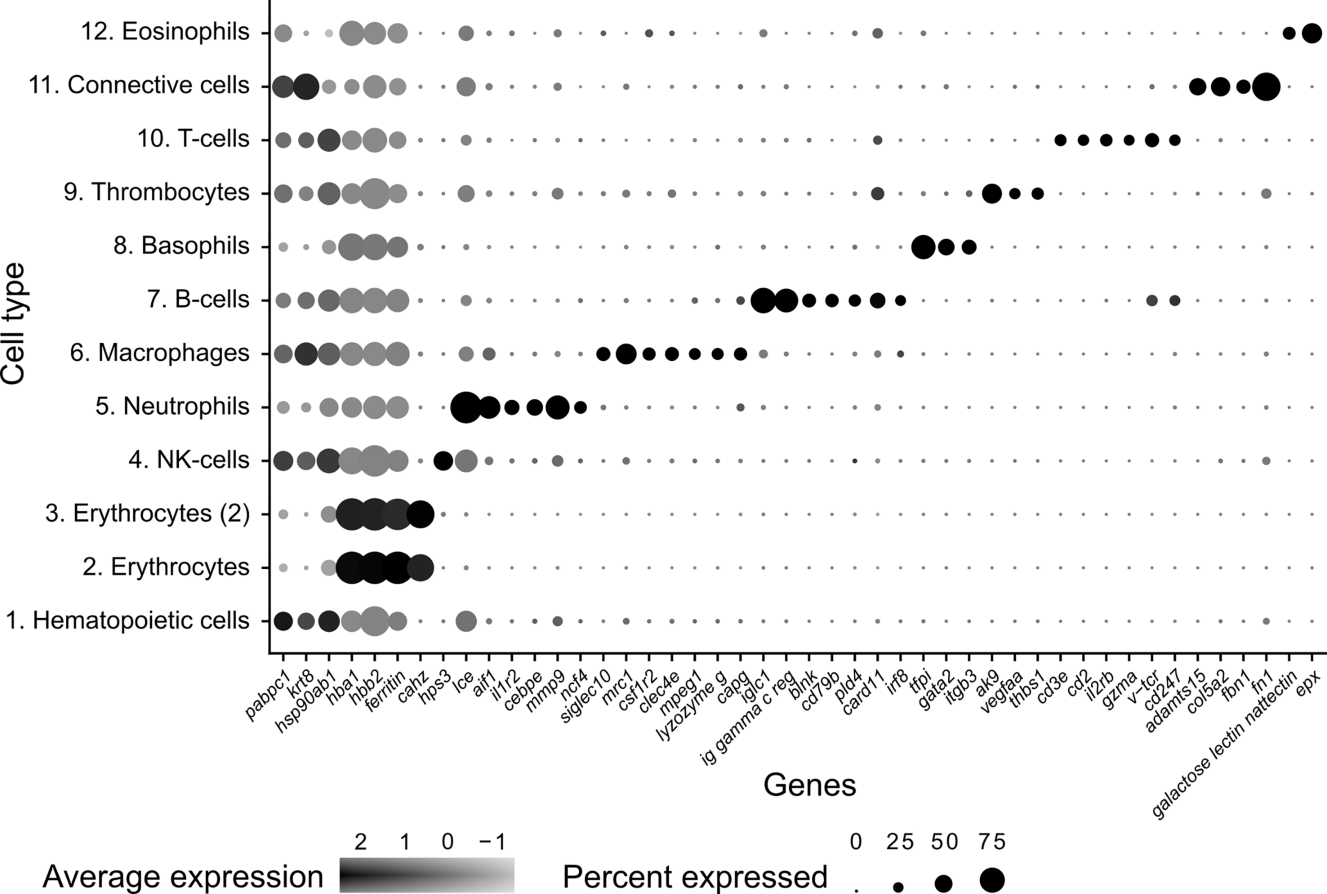
Dot plot visualization of deduced cell markers used to define cell clusters in *Syngnathus typhle*. Dot size denotes the percentage of cells expressing the gene within each cluster and the mean expression level of active expressional cells is indicated by the color intensity.

**Figure 3:**
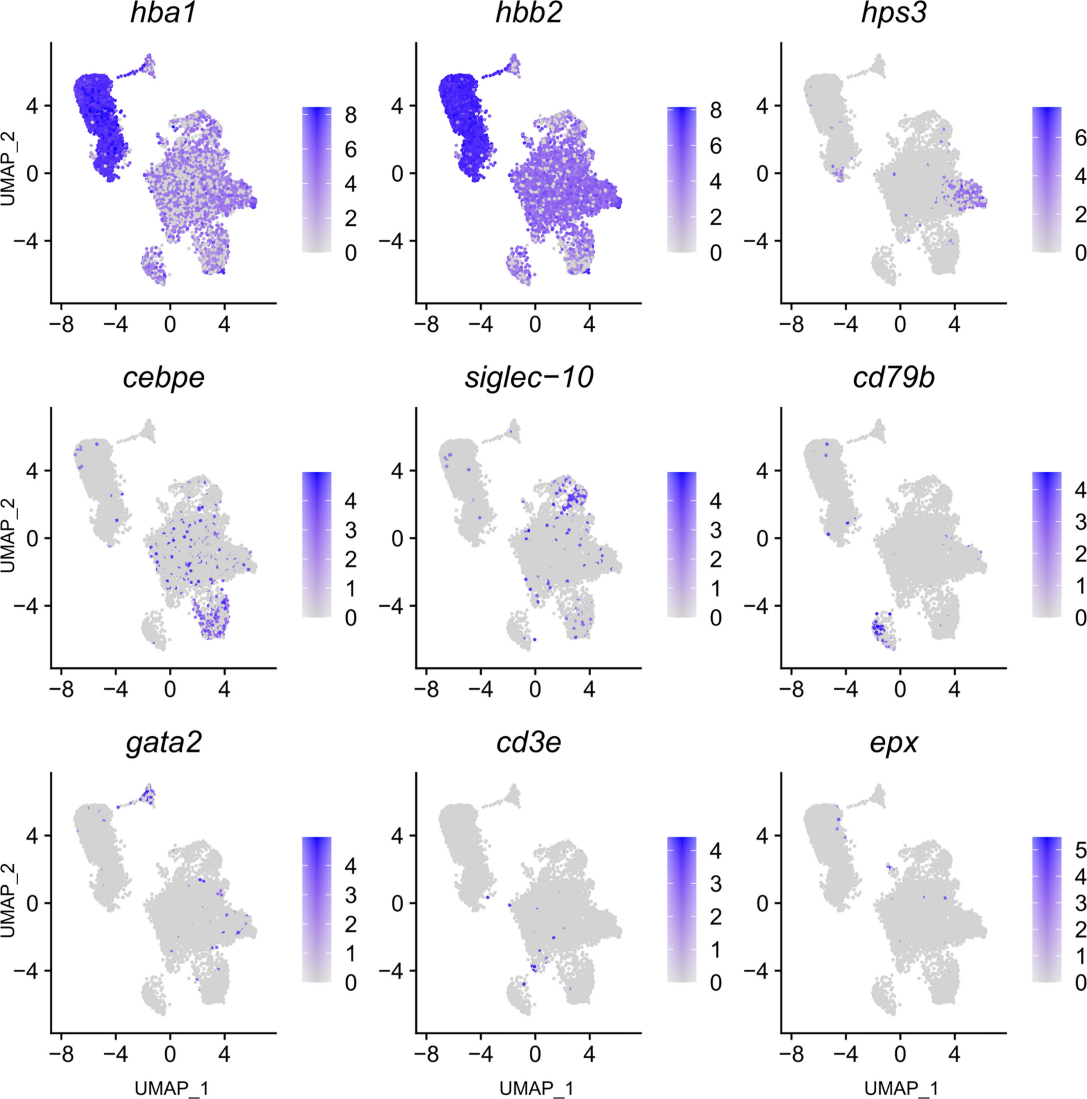
Feature plot highlighting *Syngnathus typhle* blood and head kidney cells expressing selected genes characterizing selected immune cell clusters within Uniform manifold approximation projection (UMAP). Increased cell color intensity indicates increased gene expression.

**Figure 4:**
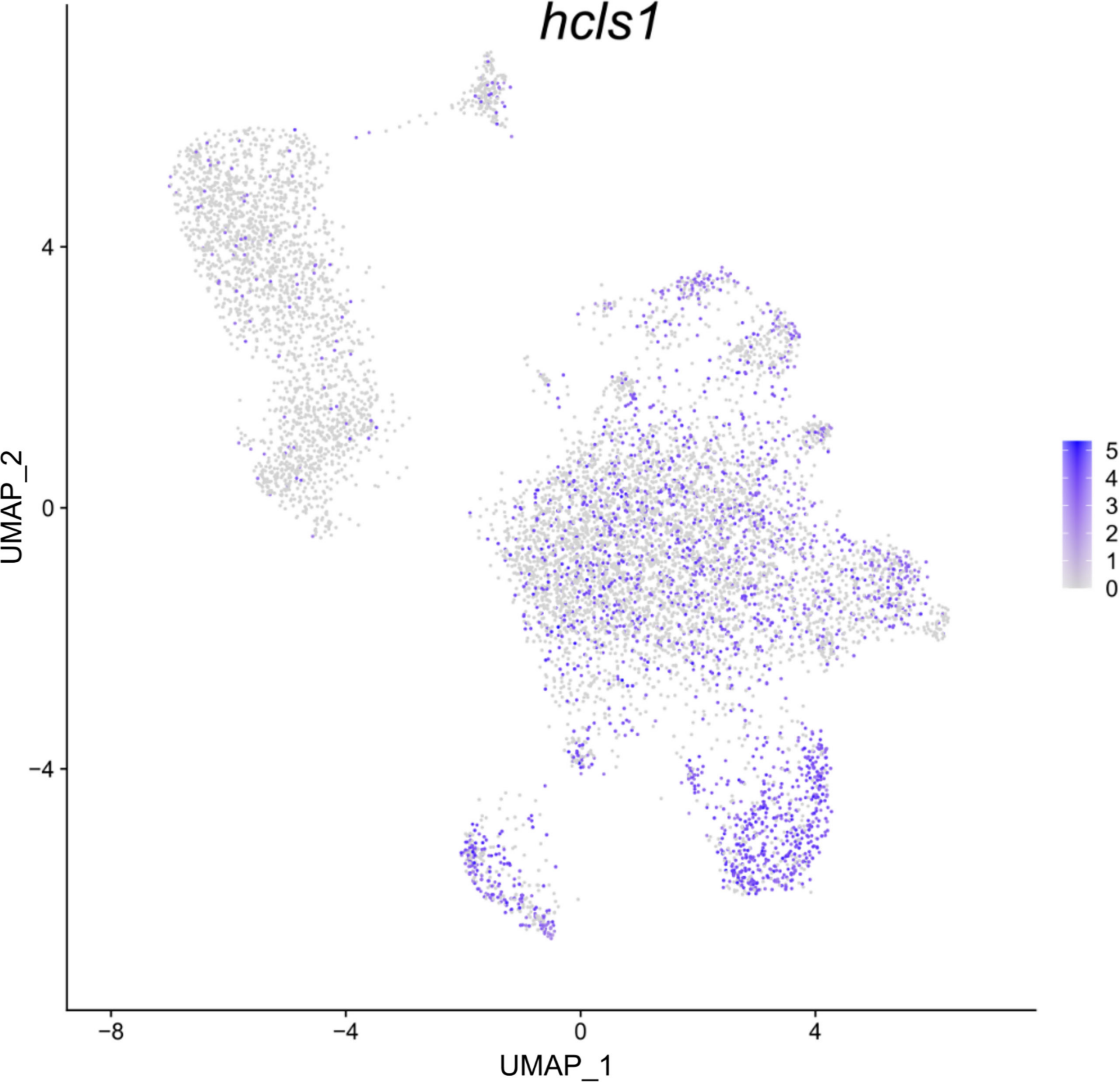
Feature plot highlighting *Syngnathus typhle* blood and head kidney cells expressing the *hcls1* gene within a Uniform manifold approximation projection (UMAP). Increased cell color intensity indicates increased gene expression.

Differential gene expression analysis provided the foundation for putative cell type identification by characterizing UMAP clusters by their significant gene expression profiles. Significant genes with immune system roles and relevant for cluster identification can be found in **Figures 2** and **3** and in the supplementary material (**Tables S2**–**13**). The putative function and classification for cells belonging to the largest cluster central to the UMAP visualization, was hematopoietic or progenitor immune cells (4,508 cells). This was owing to the lack of strong immune markers and presence of genes involved in cell cycle processes (*hsp90ab1*) and protease inhibition (*plcp1*). Additionally, two genes (*pabpc1, krt8*) have been shown to be strongly upregulated in hematopoietic cells previously (38) as well as a number of ribosomal related genes. In relation to hematopoietic cells, the hematopoietic lineage cell-specific protein (*hcls1*) was expressed significantly in three other clusters (B, T and Neutrophils) but not in the overall hematopoietic cell cluster itself, despite sporadic expression being visible within the cluster (**Figure 4**). The majority of cells making up the hematopoietic cluster could be attributed to those extracted from the head kidney (**Supplementary Figure 4**). Two closely related clusters that showed distinct separation from the other groupings were identified as erythrocytes (1,332 and 714 cells), based on their dominant upregulation of hemoglobin subunits (*hba1* and *hbb2*). Additionally, there was a distinct upregulation of ribosomal related transcripts as well as a few genes with immune related function that were identified (*h2-d1* and *ifi27*). Converse to the hematopoietic cluster, erythrocyte clusters were dominated by blood derived cells (**Supplementary Figure 4**). Lastly, one small cluster perceived to be related to structural/connective tissue (29 cells) was identified based on the strong upregulation of fibrous binding (*fbn1* and *fn1*), muscle regeneration (*adamts15*) and collagen (*col5a2*) related genes. The remaining eight clusters were assigned to different immune cell types.

### Natural Killer Cells

The largest cluster was identified as putative natural killer-like cells (NK-cells) (586 cells), largely due to the expression of *hps3*, a lysosome related protein implicated in NK-cell function (39). This cluster exhibited a reduced number of significantly differentially expressed genes when compared with all other clusters, providing fewer candidates for identification. A number of granular related serine proteases (*prss1*, *chymotrypsin b*), an immune implicated cytoskeleton regulator (*msn*) (40) and a cytoplasmic adapter associated with NK-cell receptors (*shc1*) (41) were present but at reduced significance.

### Neutrophils

The next largest cluster, which was named as the neutrophils (560 cells), provided a number of strongly upregulated immune genes and specific markers. One of the most prominent was the enzyme *lce* (egg hatching) which was previously used as a neutrophil marker in codfish (23). *Cebpe*, a gene encoding an enhancer binding protein thought to be essential for neutrophil development (42) and the neutrophil cytosol factor 4 (*ncf4*) were also highly expressed. Other indication markers include *il-1r*, which is an important neutrophil receptor (43), the inflammation factor *aif1*, which is expressed in neutrophils and monocytes (44), and the neutrophil cell migration-inducing *mmp9* (45).

### Macrophages

As with the neutrophil cluster, the perceived macrophage cluster was supported by an array of easily identifiable upregulated gene markers (364 cells). These include, *csf1r2* (macrophage differentiation), a member of a group of receptors recognized as macrophage markers in teleost fish (46–48), *mrc1* (mannose receptor) a macrophage marker in Atlantic cod and humans (23, 49), and *clec4e*, a pattern recognition receptor shown to highly expressed in macrophages (50). *Cd209*, a receptor found on antigen presenting cells which promotes MHC II presentation of the HIV virus and was one of the most upregulated genes found within the cluster. Additional gene contributors that helped define the cluster include the macrophage related protein, *mpeg1*, *lysozyme g*, and the macrophage capping protein, *capg*.

### B and T-Cell Lymphocytes

A large cluster of B-cells (265 cells) was identified by the upregulation of a high number of immunoglobulin components (e.g., *iglc1*, Ig lambda chain, *ig heavy chain*, *ig kappa chain*) which are exclusively released by B-lymphocytes. Further support was provided by the upregulation of *irf8*, *cd53* (B-cell differentiation and development), *swap-70, blnk* (B-cell activation), *card11* (B-cell signalling) and the B-cell antigen receptor *cd79b.* In addition, two T-cell receptors were among the top 20 differentially expressed genes within this cluster (*cd247* and *v-tcr*).

A population of T-cells were also strongly represented (cluster 10) (53 cells) with the upregulated expression of four universal T-cell receptors (*cd2*, *cd3e, v-tcr and cd247*), as well as the interleukin receptor subunit *il2rb.* Incidentally, IL2R has been shown to play central immune suppressive role when expressed by regulatory T (Tregs) cells (51). Lastly, the cytotoxic protease *gzma* was also highlighted for its immune related activity and previously used as a marker for cytotoxic cells in cod (23).

### Thrombocytes

Thrombocytes (61 cells), or platelets, were also represented in *S. typhle* with differentially expressed genes including *ak9*, which can influence platelet and blood coagulation in humans (52). Further confirmation is provided by the upregulation of *thbs1*, a previously labelled thrombocyte marker in codfish (23) and a prominently expressed gene in thrombocytes (53). The vascular endothelial growth factor (*vegfa*) which shares a functional relation with platelets and tissue wound healing (54) was also highly expressed within the cluster.

### Basophils and Eosinophils

A selection of basophil related genes characterized cluster 8, including *tfpi* and *itgb3* (37) (174 cells). However, the most notable upregulation came from the gene coding for GATA2 (cell differentiation), a recognized cell marker for basophil granulocytes (37, 44), distinguishing it from its close immune cell relatives.

The smallest cluster isolated in this study was attributed to eosinophils (12 cells). A large number of C-type lectins (*Galactose-specific lectin nattectin*) characterized the clusters expression profile, while the upregulated expression of eosinophil peroxidase (*epx*), a common marker and constituent of eosinophil intracellular granules in humans (55), gave further support to a small eosinophil population presence in pipefish.

## Discussion

Previous work has described the evolutionary adaptations and genomic alterations that have shaped the peculiarities within Syngnathiformes immune system, including the loss of MHC II in *S. typhle* and an increased diversification of MHC I in the syngnathids that have evolved unique male pregnancy (4, 11). The ramifications of these evolutionary changes on cellular expression level in *S. typhle* have been left unexplored up until this study, which was the first to characterize putative pipefish immune cell populations based on their individual gene expression profiles using single-cell RNA sequencing. Successful identification of a number of integral immune system constituents was achieved and the presence of relatable immune cell markers, congruent with those in other model organisms, provides a crucial baseline for future experimental and immune assessments in syngnathid fishes.

In *S. typhle* and other bony fishes, the head kidney is an important lymphoid organ and the epicentre for immune cell hematopoiesis (56–58). Appropriately, the dominance of head kidney derived cells in the perceived hematopoietic progenitor cell cluster here supports the cell type classification. The size of the hematopoietic cluster shares similarities with previous single-cell transcriptome studies on zebrafish head kidney extracted cells (59). Research on zebrafish has also shown that the progressive maturation of thrombocytes is characterized by a shift towards thrombocyte functional genes and suppression of hematopoietic related genes relevant in cell proliferation and ribosomal biogenesis (60). This could go some way to explaining the lack of upregulated specific immune cell markers within the hematopoietic cell cluster, while the close relation with most of the other more distinguished cell groups could be an indication of immature immune cells types yet to become immunologically active. Moreover, while lower in expressed significance, the upregulation of ribosomal transcripts within the hematopoietic cluster matches the ribosomal indications expressed by Macaulay, Svensson (60) and Khajuria, Munschauer (61) with regards their role in hematopoiesis and cell differentiation. Interestingly, the presence of *hcls1*, a gene upregulated in the T and B cell clusters, was also shown to be expressed in many cell contingents of the hematopoietic cell cluster. The presence of these interspersed cells could indicate that many these cells are at different stages of maturity, while its strong expression in T- and B-lymphocytes could relate to *hcls1*’s antigen signalling function described previously (62, 63).

Fish red blood cells are nucleated and have been shown to express immune related genes along with those related to gas exchange (64–66). This supports the expressed presence of *h2-d2* and *ifi27* here, suggesting erythrocytes could hold immunological relevance among *S. typhle’*s defenses. Indications suggest, that the separation of the two perceived erythrocyte clusters could be due to a batch effect stemming from the blood extracted from two different individuals. It is therefore, challenging to determine if additional factors influence the cluster differences. Erythrocyte stage of maturity for example have been reported in fish previously, with expression differences existing between immature reticulocytes and mature red blood cells (67–69).

An encouraging number of immune related genes were found in *S. typhle*, especially when identifying, T-cell, B-cell, neutrophil and macrophage populations. Managing to identify these crucial immune system constituents is promising for future syngnathid immune studies, which will allow further sub-cell type distinctions within these white blood cell groups. Neutrophils constitute the largest circulating leukocyte population in humans (50-70%) (70, 71), and while circulating neutrophil percentages in teleost fish are markedly reduced in comparison (~5%) (72, 73), head kidney neutrophil reserves have been shown to be extensive (72, 74), as was the case here in *S. typhle*. *Mmp9*, a neutrophil marker in cod and humans was suggested to drive neutrophil migration in mammals (45), and its upregulation here could be an indication of a similar influence in syngnathid fishes. One of the most convincing markers extracted from the neutrophil cluster is the neutrophil development factor *cebpe*, which has been used as a neutrophil identifier in zebrafish (75).

The strong upregulation of the MHC II associated *cd209* in the macrophage cluster, an immune receptor expressed in antigen presenting cells, poses some questions. Previously, *Cd209* expression has been identified in the MHC II devoid Atlantic cod (76, 77). This considered, it would appear that despite the loss of MHC II in both species *cd209* expression has been conserved. This could be an indication that it has adopted alternative antigen processing purpose or remains a phagocytic tool capable of facilitating viral and bacterial uptake. Macrophages are known to differentiate into pro-inflammatory (M1) and anti-inflammatory (M2) states (78). *Cd209* has been shown to be highly upregulated in M2 macrophages compared with pro-inflammatory cells in mammals (79), while M2 macrophage activity has been linked to tissue remodelling and wound healing (78, 80, 81). Therefore, the elevated presence of *cd209* here could be associated with anti-inflammatory M2 macrophages, however, determining the specific function of *cd209* in syngnathids requires further investigation.

A number of intriguing T-cell markers were featured in *S. typhle* including *gzma*. Granzyme a and -b are protease constituents of cytotoxic T-cell granules in humans (82, 83), while *gzma* in particular has been shown to be an important innate immunological component in fish (84). *Gzma* was also highlighted in codfish in association with a novel type of GATA-3 cytotoxic cell (23). Another marker, a subunit of the IL2R receptor associated with immune suppression, is an indicator for Tregs (51) and CD8^+^ cytotoxic T and NK-cell granulocytic defenses in fish (75, 85). Taken with the universal T-cell receptors represented within the cluster and the absence of additional markers, such as *gata-3* indicative of the cytotoxic cell lineage previously detected in codfish, make it difficult to conclusively allocate this group of cells to one specific T-cell subset. Nevertheless, in combination with additional immune cell isolation studies, these markers are vital when it comes to identifying T-cell subsets at a higher resolution in the future. Genomic assessments have concluded that *cd4* has been lost in *S. typhle*, bringing into question the functional or overall presence of CD4^+^ T-cells (4). Appropriately, no markers specifically associated with CD4^+^ T-cells were identified in this study, providing further support of their evolutionary disappearance in *S. typhle*. Whilst conclusively classifying the T-cell cluster presented here as CD8^+^ cytotoxic T-cells was not possible, the unearthing of a number of MHC I related pathway constituents and cytotoxic related genes supports the presence of the CD8^+^ T-cell subset in pipefish. However, determining whether the CD8^+^ T-cell subset or alternative innate immune cell types are able to offset the loss of the MHC II pathway in *S. typhle* requires further investigation.

T-cell receptors and B-cell receptors in humans were thought to be exclusively expressed by their namesakes. This traditional concept has been challenged recently by Ahmed, Omidian (86) who managed to isolate “dual expresser” lymphocytes capable of expressing both receptor types. This could explain the presence of a number of T-cell receptors that were found in both the B- and T-cell clusters. Alternatively, a small T-cell subset may be imbedded with the larger B-cell cluster, grouping together based on upregulated genes shared between the lymphocyte lineages that transcend these receptors. Further work should attempt refine and explain the cell sub-types that exist within these two integral adaptive immune cell groups.

Eosinophils, like basophils and neutrophils, are granulocyte white blood cells charged with immune surveillance and inflammatory roles in humans (87). Each are equipped with an array of cytoplasmic granules, of which some hold C-type lectins (88). The upregulated expression of eosinophil peroxidase (*epx*), a common marker and constituent of eosinophil intracellular granules in humans (55), gives convincing support to a small eosinophil population present in pipefish. Isolated initially from the venom of *Thalassophryne nattereri*, *Galactose-specific lectin nattectin* (*nattectin*) is a C-type lectin with hemagglutination activity (89). The prominent expression of *nattectin*, or a potentially similar C-type lectin within the cluster, could indicate that these granulocytes assist with coagulation or a similar immunomodulatory function that has been reported previously concerning *nattectin* (89, 90).

Deduced NK-cell-like cells in fish have been identified previously, however, clear transcript markers are still missing making their identities difficult to determine (23, 24). Characterizing the putative NK-cell-like assigned cluster in this study was challenging, due to the restricted number of highly significant upregulated genes driving cluster differentiation. The tentative NK-cell-like assignment in this species would require additional clarification from additional single-cell sequencing assessments, in order to be able to confirm with confidence its involvement in the *S. typhle* immune repertoire.

Although many of the expected immune system constituents were identifiable in this study, there were also absentees, with dendritic cells being the most notable. Dendritic cells occupy a small percentage (~0.1%) of the total circulating cells in the blood, but are more prominent in mucosal areas in humans and are crucial for linking the innate and adaptive immune systems (91, 92). Despite their perceived absence in *S. typhle* in this study, their identification in other teleost fishes (23, 93, 94) suggests that the perhaps the tissues or resolution of analyses used here was not sufficient for their discovery. Due to their small population and shared expressed markers with other immune cell types, it is likely that this sub-population is being masked within another cell cluster. The data presented here was also insufficient to distinguish between macrophages and their monocyte progenitors.

This investigation succeeded in describing the first in-depth molecular characterization of the broadnosed pipefish immune cell repertoire, utilizing single-cell transcriptomics. As with this study’s predecessors which delved into the sparsely explored realm of teleost immune cell populations (23–25, 27, 28), a number of key immune cell sub-populations were identifiable providing some insight into the putative immune function of *S. typhle*. Establishing a baseline expression profile for each immune cell group identified here, along with corroborated gene markers, will be crucial for future experimental work such as those carrying out infection assessments on syngnathid fishes. This molecular assistance should extend to investigations concerning *S. typhle* relatives such as seahorses and other pipefishes, with potential scope for future comparative, multispecies single-cell assessments that can lay down a comprehensive immune cell overview of the Syngnathiforme lineage. Understanding the cellular repercussions and adaptations that may have evolved within these immunologically bizarre fishes could support future medical practices by shedding light on the way certain immune cell lineages evolve.

## Data Availability Statement

The datasets presented in this study can be found in online repository. The names of the repository/repositories and accession number(s) can be found below: https://www.ncbi.nlm.nih.gov/, PRJNA781832.

## Ethics Statement

The animal study was reviewed and approved by the Ministerium für Energiewende, Landwirtschaft, Umwelt, Natur und Ditgitalisierung (MELUND) (Permit number V 242 – 57983/2018) and are in accordance with German animal welfare law.

## Author Contributions

OR, JP, and SJ conceived the study. JP and NG processed fish cell samples and analyzed the data. JP and OR wrote the manuscript with input from all co-authors. All authors contributed to the article and approved the submitted version.

## Funding

This project was supported by funding from the German Research Foundation (RO-4628/4-2) and from the European Research Council (ERC) under the European Union´s Horizon research and innovation program (MALEPREG: eu-repo/grantAgreement/EC/H2020/755659) to OR. Sequencing library creation and RNA sequencing was performed at the Norwegian Sequencing Centre, University of Oslo (www.sequencing.uio.no), Norway.

## Conflict of Interest

The authors declare that the research was conducted in the absence of any commercial or financial relationships that could be construed as a potential conflict of interest.

## Publisher’s Note

All claims expressed in this article are solely those of the authors and do not necessarily represent those of their affiliated organizations, or those of the publisher, the editors and the reviewers. Any product that may be evaluated in this article, or claim that may be made by its manufacturer, is not guaranteed or endorsed by the publisher.
