# Supplementary material for: Characterization of Pipefish Immune Cell Populations Through Single-Cell Transcriptomics
Source: Front Immunol. 2022 Jan 27;13:820152. doi: 10.3389/fimmu.2022.820152 (PMC8828949; doi:10.3389/fimmu.2022.820152)
Supplement: Supplementary file 1 [file DataSheet_1.pdf]

SUPPLEMENTARY FIGURES

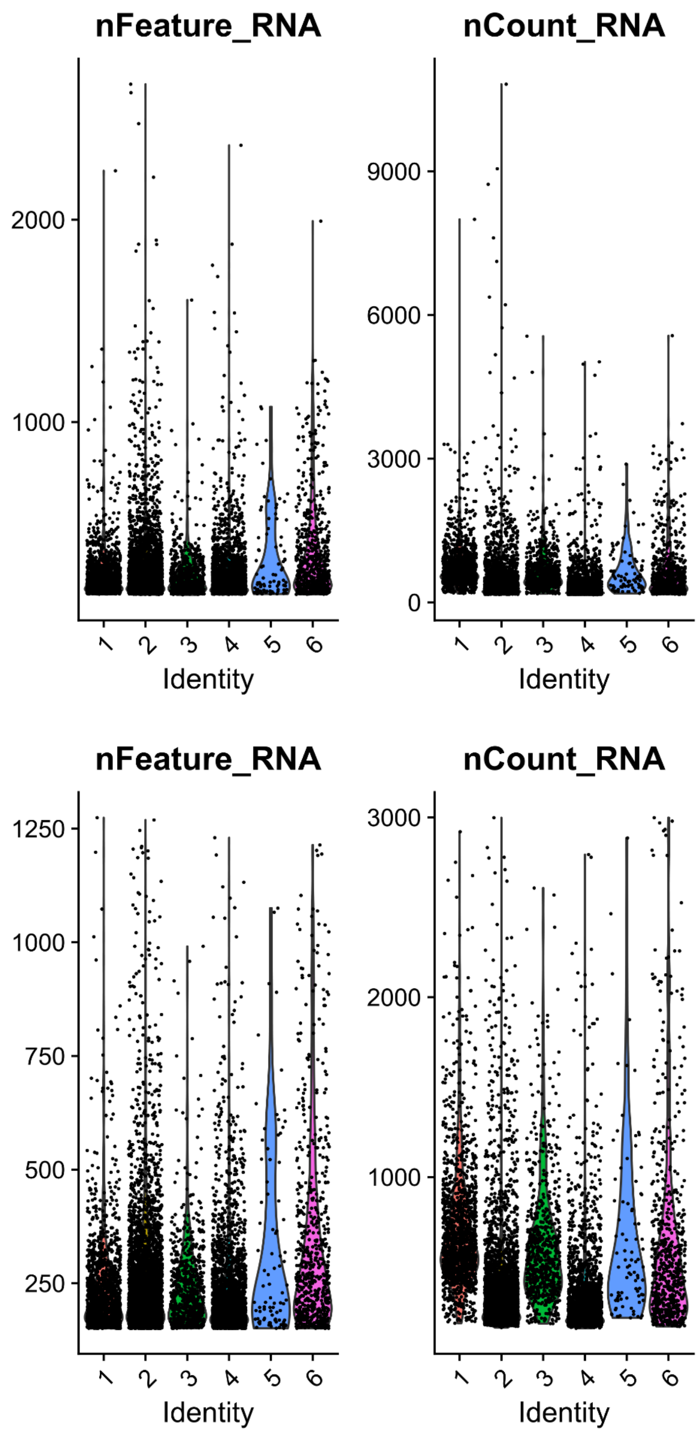

Figure 1. Violin plots showing all 6 samples before (top) and after (bottom) quality control cut offs and filtering.

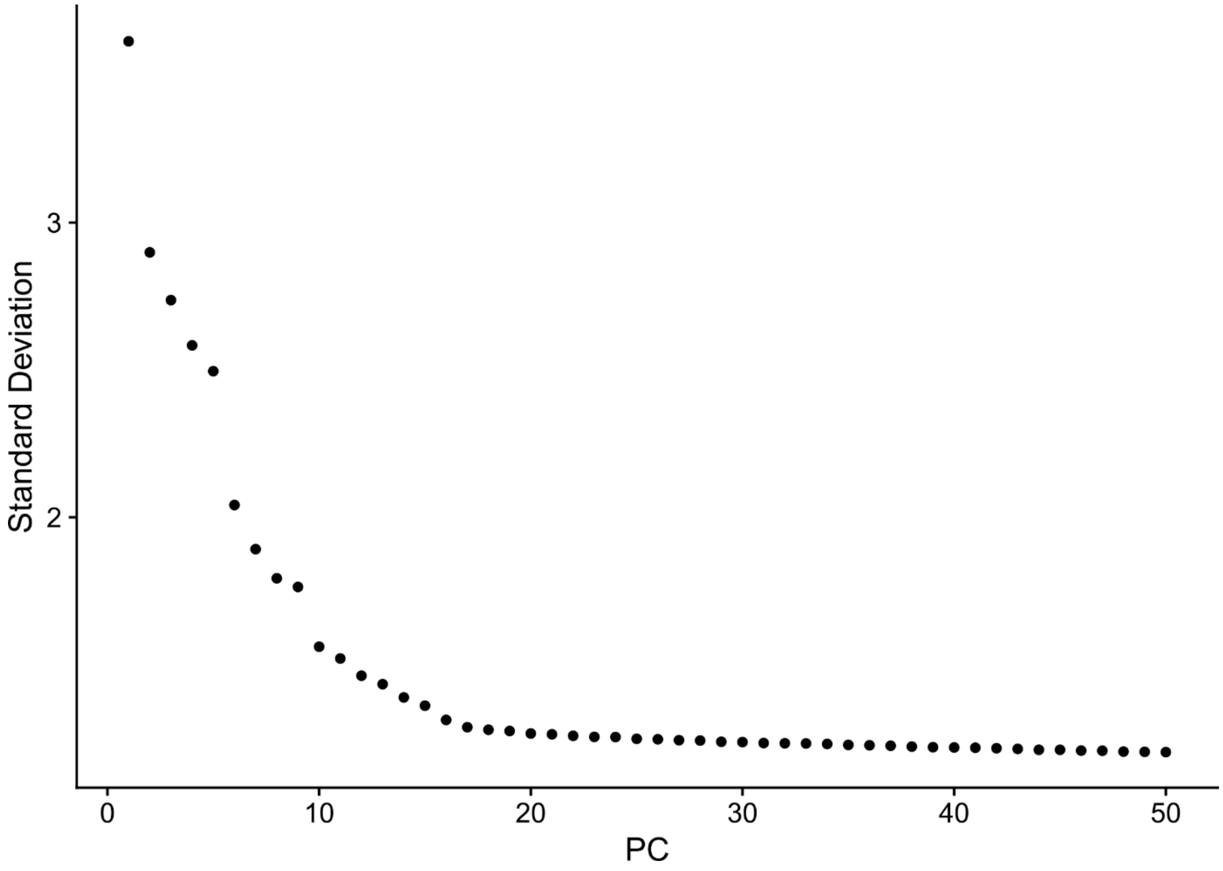

Figure 2. Elbow plot exhibiting principal component rankings based on associated explained variation.

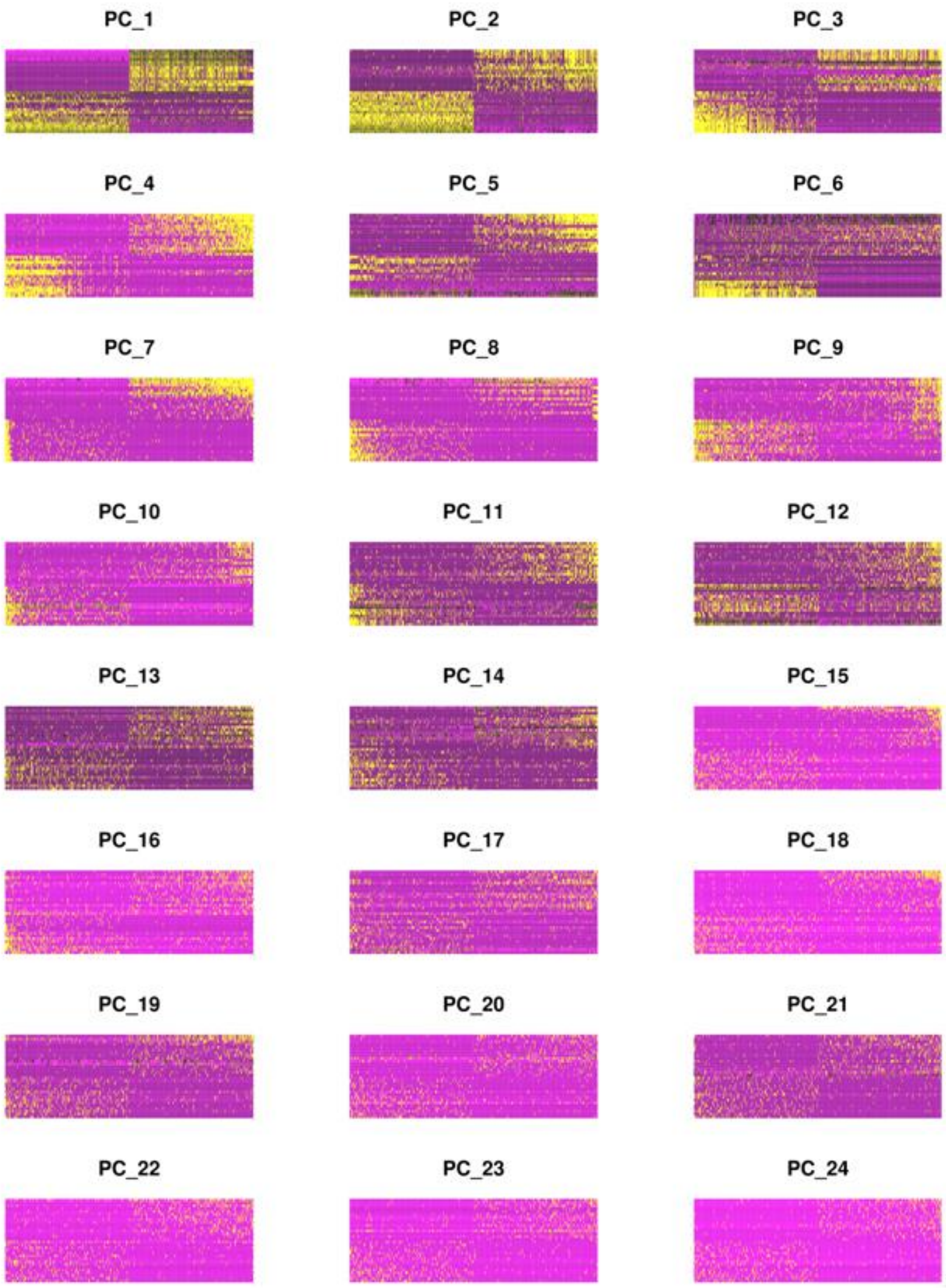

Figure 3. Differential gene expression heatmaps of the first 24 principle components, with genes representing rows and cells representing columns.

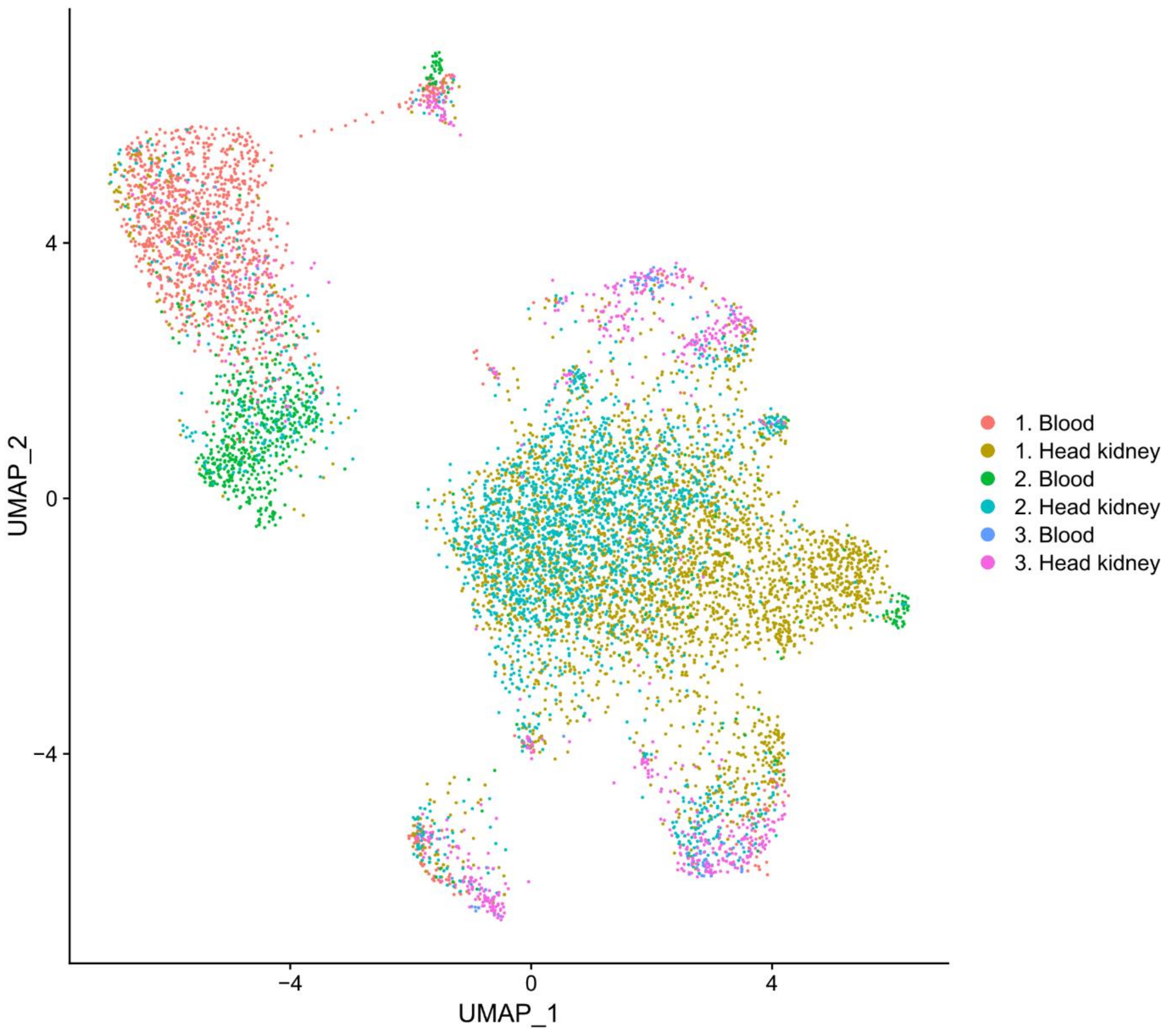

Figure 4. (a) Uniform manifold approximation projection (UMAP) showing the extracted origin of cells, from three *Syngnathus typhle* individuals. Legend numbers (1, 2, 3) indicate the fish individual.
